# Calorie labelling regulations in England: menu change not behaviour change?

**DOI:** 10.1017/S1368980025100682

**Published:** 2025-08-12

**Authors:** Alexandra Kalbus, Chiara Rinaldi

**Affiliations:** Department of Public Health, Environments and Society, London School of Hygiene & Tropical Medicine, London, UK

**Keywords:** Calorie labelling, Food policy, Menu labelling, Out-of-home food sector

On 6^th^ April 2022, legislation came into effect in England, which requires food businesses such as restaurants and takeaways with 250 employees or more to show the energy content (in calories (kcal)) of food and drink ready for immediate consumption^(1)^. Calorie disclosure is compulsory at the point of choice, including on physical menus, boards and digital menus on food delivery platforms. These calorie labelling regulations aim to reduce energy intake from foods and drinks prepared away from home. The legislation, first announced in the 2020 Tackling Obesity Strategy^(2)^, follows similar national and state-level calorie labelling policies in the USA, Canada, Ireland, South Korea and several other countries^(3)^, as well as previous voluntary strategies which achieved limited success^(4)^. Calorie labelling is hypothesised to reduce out-of-home energy intake via two mechanisms: The first is via a change in consumer behaviour, whereby the access to calorie information prompts individuals to make a different, and on average, lower-calorie, food choice. The second pathway is via a change in the calories offered, whereby the requirement to display calories encourages businesses to voluntarily lower the calorie content of food and drink items on their menus.

Evaluations of calorie labelling interventions, most of which took place in the USA, predominantly focus on the first mechanism. A recent systematic review and meta-analysis found a reduction of 11 kcal per selected meal, corresponding to –1·8 %, as a result of calorie labelling^(5)^. However, emerging research from the evaluation of the calorie labelling regulations in England finds no evidence of changes in calories purchased: Polden *et al.* ^(6)^ estimated changes in calories ordered and consumed in a pre-post customer intercept design, while Luick *et al.* ^(7)^ used an interrupted time-series design to analyse changes in calories purchased in workplace cafeterias. Both observed no impact of calorie labelling on consumer behaviour.

A framework suggested by Adams *et al.* ^(8)^ may help explain the policy’s limited impact on consumer behaviour. Accordingly, public health interventions can be categorised along the continua: individual to population and low- to high-agency approaches. Greater effectiveness and more equitable effects are expected for population-level interventions that require low effort and engagement from individuals (e.g. lowering the sugar content in soft drinks) as compared with those targeting specific individuals and requiring high levels of agency (e.g. distributing information on healthy diets for children to parents)^(8)^. Consumer behaviour change as a consequence of calorie information provision, while population-wide, requires considerable ‘agentic demand’ from consumers who must notice the calorie information and use cognitive resources to interpret and act on the intervention (in the context of external socio-cultural, physical-environmental and financial cues)^(9)^. An analysis from the International Food Policy Study found that in late 2022, only 26 % of customers in England noticed calorie labels, while only 14 % used them to make their food choice^(10)^.

Menu change, including reformulation and the introduction of lower-calorie items, on the other hand, constitutes a population-wide intervention that requires relatively little agency from consumers. Hence, from a theoretical perspective, menu changes may be a more effective and equitable intervention than information provision. Investigations into this mechanism suggest that calorie labelling may lead to small decreases in overall calories offered by businesses. A meta-analysis by Zlatevska *et al.* ^(11)^ found an average reduction of 15 kcal per meal due to changes in menus following the announcement and implementation of calorie labelling legislation in the USA. In the UK, a study of seventy-eight restaurant chains in the UK found a mean reduction of 9 kcal per menu item between September 2021 and September 2022^(12)^. In their analysis of workplace food offer and purchases, Luick *et al.* ^(7)^ also observed that the mean calorie content of menu options decreased over time.

To determine if calorie labelling regulations incentivise menu changes, we need to understand how menus change as a consequence of menu labelling, as a uniform calorie reduction is unlikely. Previous research from the USA and UK indicates that changes to menus, i.e. introducing lower-calorie items and removing higher-calorie items, drive overall calorie reduction rather than reformulation of items continuously on menus^(12–15)^. Research from the UK and North America indicates that the extent of menu changes varies by menu section, with greatest reductions generally observed in main dishes^(12,13)^, while other research did not observe differences by menu category^(15)^. Generally, no reduction in calories was identified among items in the children’s menu section^(11)^. The evidence on whether calorie reduction following mandatory calorie disclosure differs by restaurant type is mixed, with some studies reporting greater reductions among sit-down compared with fast-food restaurants^(16)^, and others finding no difference^(15,17)^. The only study to explore changes in calorie content following England’s calorie labelling regulations documented a mean reduction of 38 kcal (95 % CI –42, –5) per menu item for sit-down restaurants and a 42 kcal (95 % CI 27, 57) increase among fast-food outlets^(12)^. In addition, an analysis by Bleich *et al.* ^(13)^ suggests that calorie reductions tend to be greater among non-core items in restaurants with a specific focus, e.g. items other than pizza in a pizza restaurant. Analyses of calorie reductions in specific food items (e.g. pizza, burgers and sandwiches) similarly report mixed results^(12,14,18,19)^.

Menu changes constitute fruitful venues for further research on the effectiveness of the calorie labelling regulations in England. Once menu changes are ascertained, it is important to understand who is most likely to be affected, and in case of calorie reductions, benefit from them. For example, this could be explored by analysing consumer expenditure data to determine if some population groups, for example, by age, region of residence or socio-economic status, are more likely than others to purchase, and consume items with (greater) calorie reductions. While research into the consumer behaviour change mechanism of calorie labelling found no differential impacts of the policy^(5,20)^, there may still be inequalities in the effectiveness of calorie labelling if menu calorie reductions affect some population groups more than others.

More research is needed to assess whether calorie reduction efforts in the out-of-home food sector may be more effective and equitable if targeted at driving menu change rather than influencing consumer food purchases. The authors of this commentary are involved in a research project that will analyse menu changes in categories co-determined with members of the public as well as local and national government to ensure they are relevant to the public and policy (https://nihrsphr.link/CARO). While not exhaustive, the case studies in this research project will begin to answer the questions of whether, and how, calories on menus changed and who is likely to benefit. Further research can build on this work and comprehensively map trends in menu changes following calorie labelling and estimate differential subsequent health impacts among the population. This will help inform stakeholders tasked with the implementation, continuation and adaptation of the calorie labelling regulations. Of particular interest will be strategies to encourage calorie reduction on menus through incentivising menu change, for example through a calorie-based levy akin to the Soft Drinks Industry Levy^(21)^. Such a re-framing of the policy, which would shift responsibility away from individuals to food businesses, could present an opportunity for more equitable improvements to the food environment.
